# Circumscribed Interest Modulates Attention to Eyes in Boys With and Without Autism Spectrum Disorder

**DOI:** 10.3389/fpsyt.2021.627365

**Published:** 2021-07-27

**Authors:** Qiandong Wang, Sio Pan Hoi, Ci Song, Tianbi Li, Cheuk Man Lam, Yuyin Wang, Li Yi

**Affiliations:** ^1^Beijing Key Laboratory of Applied Experimental Psychology, National Demonstration Center for Experimental Psychology Education (Beijing Normal University), Faculty of Psychology, Beijing Normal University, Beijing, China; ^2^School of Psychological and Cognitive Sciences & Beijing Key Laboratory of Behavior and Mental Health, Peking University, Beijing, China; ^3^Institute of Psychology, Chinese Academy of Science, Beijing, China; ^4^Department of Psychology, Sun Yat-sen University, Guangzhou, China

**Keywords:** autism spectrum disorder, eye-avoidance, circumscribed interests, visual attention, eye movement

## Abstract

Children with autism spectrum disorder (ASD) exhibit abnormal visual attention, such as diminished attention to eyes and enhanced attention to high-autism-interest objects. We tested whether high-autism-interest objects would modulate the attention to eyes in boys with ASD and typically developing (TD) boys. Twenty-two ASD and 22 TD children were presented simultaneously with human eyes and high/low-autism-interest objects (HAI/LAI) while their eye movements were recorded. We found that visual preference for eyes was influenced by competing objects in children with and without ASD. Specifically, both children with and without ASD showed reduced overall and first looking preference when eyes were paired with HAI objects relative to LAI objects. Children with ASD also showed reduced sustained viewing preference to the eyes after first looking at the eyes and late looking preference to the eyes after first looking at the objects in the HAI condition than the LAI condition, but these effects were absent in the TD group. Our study not only helps us understand some factors that impact attention to eyes, but also has implications for interventions aiming at improving eye contact in children with ASD.

## Introduction

Autism spectrum disorder (ASD) is a neurodevelopmental disorder characterized by two main core symptoms—social communication deficits and restricted interests and repetitive behaviors (1). Most previous work has focused on one of these two symptoms, while only a few studies have focused on the relation between these two core symptoms (2). Of these studies, some found an intrinsic link between social communication deficits and repetitive behaviors (3–6), while others claimed independence of the two symptoms (7–9). Considering that there are distinctive subtypes of social communication deficits and repetitive behaviors in ASD, the question that whether the two core symptoms are related is not clear (10). The current study aims to explore this relationship in children with ASD using eye movements, viewed from the visual attention point.

The two core symptoms of ASD may be reflected in the visual looking time. For instance, along with socio-communicational deficits, many eye-tracking studies have explored whether people with ASD look less at eyes than typically developing (TD) people. Although findings from these studies are mixed (11–13), a recent meta-analysis of eye-tracking studies found a significant reduction in attention to eyes in individuals with ASD than TD people (14). On the other hand, people with ASD are found to be interested in certain types of objects, referenced to circumscribed interests (CI), or high-autism-interest (HAI) stimuli (e.g., vehicles, computers, and repetitive movements) (14–17). These stimuli have been found to attract visual attention (15, 16, 18), activate neural reward circuitry (19), and induce pleasure responses (20) in people with ASD. Based on these findings, HAI objects are proposed to negatively affect the social development of people with ASD by occupying their visual attention and depriving them of sufficient social interaction experiences (16, 21). However, the relationship between visual attention to eyes, which plays an essential role in social interaction, and visual attention to HAI objects has received almost no empirical attention. From both theoretical and practical perspectives, there are significant implications of knowing whether attention to eyes in people with ASD would be improved if some factors are manipulated, such as presenting HAI or low-autism-interest (LAI) objects. Therefore, the current study aimed to investigate how HAI objects would modulate the attention to eyes in children with ASD.

In order to examine this modulation effect, we used the preferential looking paradigm by pairing human eyes with two types of competing stimuli, that is HAI or LAI objects, to explore visual preferences for eye region in children with and without ASD. Some studies have already used a similar paradigm to study the modulation effect of competing non-social objects on social stimuli like faces [e.g., (15, 16)]. However, this has been achieved via a less fine distinction between the early and late attentional components. As a result, it remains unclear whether the modulation effect is due to the consistent impacts over time or from local effects confined to particular times.

Our study expected that including HAI objects would increase the general interest level to this type of object and reduce the total looking time to eyes in children with ASD. This overall eye-preference index could reflect some combinations of initial orienting to and subsequent maintenance with stimuli, as well as looking time after initial orienting. In order to separate these components, we calculated prioritization index, sustained index, and late eye-preference index (see Data Analysis section for details) to test whether the influence of HAI on the attention to eyes was mainly driven by different mechanisms of attention. Rather than focusing simply on which object receives the greater amount of viewing time, the prioritization index indicates which object receives attentional priority in the scene. In daily life, the social meaning of eyes should be perceived very rapidly. Therefore, prioritizing for processing eyes is helpful for a successful social interaction. Additionally, the sustained index examines a sustained viewing time in the initial detection of an object. This index can reflect a general interest in an object. If people are interested in an object, they examine it longer after looking at it. Finally, the late eye-preference index examines total eye-looking time after first looking at or initial orienting to objects, but not total eye-looking across time. It may reflect a late attentional component, which is less likely to be influenced by low-level stimulus properties than an early component like prioritization index (see detailed discussions in the Discussion section). Therefore, this late eye-preference index is better than the overall eye-preference index to reflect top-down attention to the eyes.

## Method

### Participants

After excluding children with ASD who had a low IQ (IQ <70, measured by Wechsler Intelligence Scale), 22 high-functioning boys with ASD and 22 age and IQ-matched TD boys from China were included in the current study. Sample size was determined by availability and was greater than or equal to that of other studies using a similar paradigm [e.g., (15, 16, 22)]. Furthermore, when we opted for a moderate effect size ($\eta_{\text{p}}^{2}$ = 0.06), 0.8 power, an alpha of 0.05, and 0.5 as correlation among repeated measures to perform power analysis by using G^*^Power software (23), a total sample of at least 34 individuals is required by a repeated measures analysis of variance (ANOVA) with Group (ASD and TD) as the between-subjects factor, and Condition (HAI and LAI) as the within-subjects factor.

We recruited boys only in our study to control for gender differences in interest in objects (22). The children with ASD were all previously diagnosed by professional pediatricians in licensed hospitals according to the criteria of ASD in DSM-V (1), and were further confirmed by using the Autism Diagnostic Observation Schedule [ADOS; (24)] and the Autism Diagnostic Interview-Revised [ADI-R; (25)]. The TD children were recruited from a typical primary school, and the teachers and parents reported no concern about any potential developmental or psychiatric disorder. All participants had normal or corrected to normal vision with no color-blindness. Table 1 shows the participants' characteristics.

**Table 1:** Characteristics of the participants.

	**ASD (** ***N*** **= 22)**		**TD (** ***N*** **= 22)**		***t***
	***M***	***SD***	***M***	***SD***	
Age (years)	7.01	1.32	7.51	0.65	−1.59
Full Scale IQ^†^	100.95	17.29	96.41	10.50	1.05
ADOS Total Severity	8.86	1.39	–	–	–
SA Severity^‡^	8.73	1.42	–	–	–
RRB Severity^§^	8.27	0.94	–	–	–
ADI-R			–	–	–
Social Interaction	22.09	5.18	–	–	–
Communication	17.73	4.43	–	–	–
RRB	9.09	2.02	–	–	–
D Scale^¶^	3.45	1.22			

† *IQ was measured using the Chinese abbreviated version of Wechsler Intelligence Scale for Preschool and Primary Children-Forth Edition (26), or Chinese abbreviated version of Wechsler Intelligence Scale for Children-Forth Edition (27)*;

‡ *SA Severity = ADOS Social Affect Severity*;

§ *RRB Severity = ADOS Restricted, Repetitive Behavior Severity; SA and RRB Severity were calculated according to Gotham et al. (28)*;

¶ *D Scale is abnormality of development evident at/before 36 months*.

This research was conducted according to the ethical standards laid down in the 1964 Declaration of Helsinki and was approved by the Ethical Committee of the sponsoring university. We obtained all of the children's oral consent and their parents' written consent before the onset of the experiment.

### Materials

**Figure 1:**
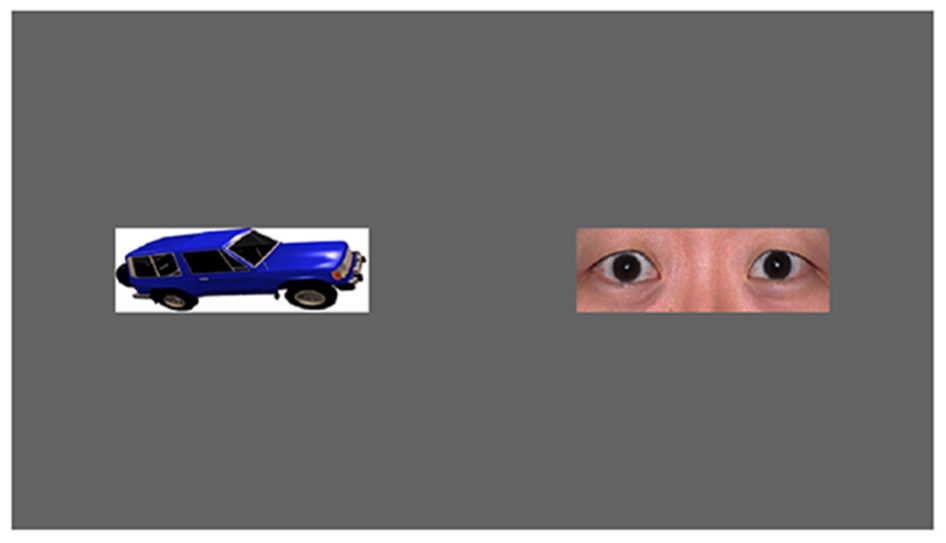
Sample stimuli in the HAI condition.

The stimuli consisted of 24 images depicting the eye region cropped from different faces displaying fear expressions, which were extracted from the NimStim Face Stimulus Set (29), a free and widely used database [e.g., (30, 31)]. Fearful eyes were used as stimuli, given previous evidence that eye avoidance in ASD was most likely to occur when scanning threatening facial expressions (18). HAI object images included 12 different cars, and LAI object images included 12 different vegetables (15, 16). There were a total of 24 trials presented on a 21″ monitor. In each trial, one eye image was randomly paired with one HAI or one LAI image, resulting in 12 paired eyes and HAI object images (HAI condition), and 12 paired eyes and LAI object images (LAI condition) (Figure 1). The allocation (left/right) of stimuli within a trial was counterbalanced. Each image subtended a visual angle of 12×4° to the children.

### Procedure

Children sat ~60 cm away from a monitor. Eye movements were recorded by a Tobii Pro X3-120 eye tracker (Tobiitech Technology, Stockholm, Sweden; sampling rate: 120 Hz) during the whole experiment. Before the formal experiment, children were asked to pass a five-point calibration procedure. The calibration process was repeated when necessary until both eyes achieved good mapping on all five test positions (smaller than 1° visual angle).

In each trial, an attention-getter (a cartoon character subtending a visual angle of 4×4°) was first presented in the center of the screen, which disappeared when children's gaze was detected within the cued region. Then, one of the 24 paired images was presented for 3,000 ms, and children were asked to view it freely. The stimuli were presented in a randomized order with the constraint that the same condition and same eye position could not occur more than three in a row.

### Data Analysis

#### Data Preprocessing

Trials with more than 30% missing gaze data (i.e., low-quality data defined as validity codes > 1 from Tobii raw data) were considered unreliable and excluded from the analysis [e.g., (32)] to ensure the quality of data, which was stricter than some previous studies (33, 34). Missing gaze data that had a gap shorter than 75 ms in the other trials were filled in using linear interpolation based on the last valid sample before the missing gap and the first valid sample after the gap. Average gaze positions of the left and right eyes were used to calculate fixations based on I-VT fixation filter (35) with the following parameters settings: (1) the velocity threshold was set at 30°/s; (2) Fixations close spatially and temporally (<0.5°, <75 ms) were merged to prevent longer fixations from being separated into shorter fixations because of data loss or noise; (3) Fixations shorter than 60 ms were discarded. Areas of interest (AOIs) were defined around the eyes and objects. In our analysis plan, trials would be further discarded if no fixations were detected in the two AOIs. However, no trials were discarded according to this criterion. The valid trial number was similar for the ASD (*M* = 22.50, *SD* = 2.44) and TD (*M* = 23.55, *SD* = 1.47) groups, *p* > 0.05.

#### Main Eye Movement Indices

Preliminary analysis revealed that the ASD group (*M* = 1.52 s, *SD* = 0.48 s) looked less at the two AOIs than the TD group (*M* = 2.07 s, *SD* = 0.52 s), *t*_(42)_ = −3.62, *p* = 0.001, Cohen's *d* = 1.09. Thus, in our primary analysis, in order to quantify the overall visual preference to the eye image relative to the object image, we converted total fixation data into proportion data. Using proportion data was also consistent with previous studies using the preferential looking paradigm (15–17). We calculated the *overall eye-preference index*, defined as the proportional total looking time on the eye AOI against the total looking time on both the eye and object AOIs. A well-above-chance level (50%) *eye-preference index* represents an overall looking preference for the eyes over the objects. We further calculated a *prioritization index*—frequencies of the first look at the eyes against the total valid trials as an initial orienting component of attention. A well-above-chance level of *prioritization index* represents a first preference for the eyes over the objects. Besides, we calculated the *sustained index*. First, we calculated the sum of the duration of all fixations of the first entry to the eye or object AOI, until a child shifted attention away from the AOI (sustained duration). The *sustained index* is defined as sustained duration on the eye AOI against the sum of sustained duration on the eye and object AOIs. This index represents maintaining of engagement with eyes after initial orientation relative to the object. Finally, *the late eye-preference index* was very similar to the *overall eye-preference index*. It was calculated as the proportional total looking time on the eye AOI against the total looking time on both the eye and object AOIs after first looking at an object AOI, but not the whole trial duration. The calculation of these eye movement indices can be referred to Holmqvis et al. (36).

## Results

### Overall Eye-Preference Index

**Figure 2:**
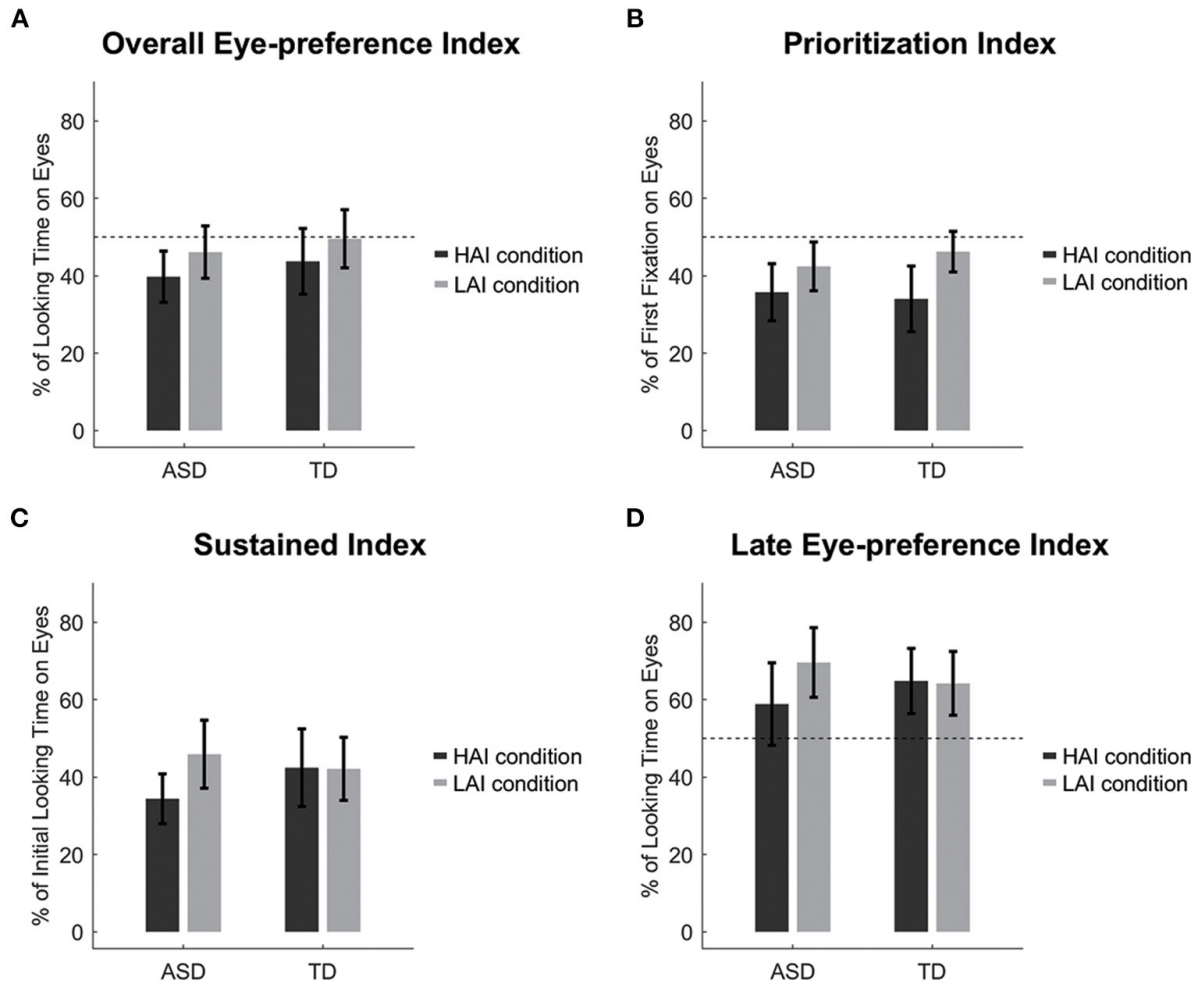
Eye movement results. (**A**) Overall eye-preference index; (**B**) Prioritization Index; (**C**) Sustained index; (**D**) Late eye-preference index. Dashed lines represent the chance level and error bars represent standard error.

A 2 (Group: ASD and TD) × 2 (Condition: HAI and LAI) repeated measures ANOVA was conducted. Only the main effect of Condition was significant, *F*_(1, 42)_ = 13.05, *p* = 0.001, $\eta_{\text{p}}^{2}$ = 0.24. Both groups showed decreased attention to eyes when HAI objects were present compared to when LAI objects were present (Figure 2A). The main effect of Group and the Group × Condition interaction effect were all not significant, *F*_(1, 42)_ = 0.54, *p* = 0.466, $\eta_{\text{p}}^{2}$ = 0.013, and *F*_(1, 42)_ = 0.02, *p* = 0.876, $\eta_{\text{p}}^{2}$ < 0.001, respectively.

We also compared the *eye-preference index* to the chance level by using a one-sample *t-*test with Bonferroni correction (alpha level = 0.0125 for four times comparison). Children with ASD showed a decreased looking preference for the eyes over the HAI objects, *t*_(21)_ = −3.03, *p* = 0.006, Cohen's *d* = 0.65, but not LAI objects, *t*_(21)_ = −1.13, *p* = 0.272, Cohen's *d* = 0.24. TD children showed no significant results, *t*_(21)_ = −1.45, *p* = 0.162, Cohen's *d* = 0.31, and *t*_(21)_ = −0.12, *p* = 0.907, Cohen's *d* = 0.03, for HAI and LAI objects, respectively.

### Prioritization Index

Like the *eye-preference index*, only the main effect of Condition was significant, *F*_(1, 42)_ = 8.65, *p* = 0.005, $\eta_{\text{p}}^{2}$ = 0.171. Both groups showed a reduction in the first preference for the eyes when HAI objects were present compared to when LAI objects were present (Figure 2B). The main effect of Group and the Group × Condition interaction effects were all not significant, *F*_(1, 42)_ = 0.07, *p* = 0.787, $\eta_{\text{p}}^{2}$ = 0.002, and *F*_(1, 42)_ = 0.74, *p* = 0.395, $\eta_{\text{p}}^{2}$ = 0.017, respectively.

We then compared the *prioritization index* to the chance level, and found that both groups showed a decreased first looking preference for the eyes over the HAI objects, *t*_(21)_ = −3.80, *p* = 0.001, Cohen's *d* = 0.81, and *t*_(21)_ = −3.70, *p* = 0.001, Cohen's *d* = 0.79, for the ASD and TD groups respectively, but not LAI objects, *t*_(21)_ = −2.36, *p* = 0.028, Cohen's *d* = 0.50, and *t*_(21)_ = −1.42, *p* = 0.171, Cohen's *d* = 0.30, for the ASD and TD groups, respectively.

### Sustained Index

A 2 (Group: ASD and TD) × 2 (Condition: HAI and LAI) repeated measures ANOVA was conducted. Note that one TD child's first looks were all directed to the object in the HAI condition, thus the sustained index in the HAI condition was not available for this child. The main effect of Group was not significant, *F*_(1, 41)_ = 0.17, *p* = 0.680, $\eta_{\text{p}}^{2}$ = 0.004. The main effect of Condition was significant, *F*_(1, 41)_ = 7.90, *p* = 0.008, $\eta_{\text{p}}^{2}$ = 0.162. Importantly, the Group × Condition interaction was also significant, *F*_(1, 41)_ = 7.22, *p* = 0.010, $\eta_{\text{p}}^{2}$ = 0.150. Simple effect analysis revealed that children with ASD had lower *sustained index* in the HAI condition than that in the LAI condition, *F*_(1, 41)_ = 15.48, *p* < 0.001, $\eta_{\text{p}}^{2}$ = 0.274, but TD children had a similar *sustained index* in the HAI and LAI conditions, *F*_(1, 41)_ = 0.01, *p* = 0.931, $\eta_{\text{p}}^{2}$ <0.001 (Figure 2C).

### Late Eye-Preference Index

A 2 (Group: ASD and TD) × 2 (Condition: HAI and LAI) repeated measures ANOVA was conducted. Note that one ASD child and one TD child did not look at the eye and object AOIs after initial orientation to the object in the LAI or HAI condition. Thus, the late eye-preference index was not available for these two children. The main effect of Group was not significant, *F*_(1, 40)_ = 0.01, *p* = 0.930, $\eta_{\text{p}}^{2}$ < 0.001. The main effect of Condition was marginally significant, *F*_(1, 40)_ = 3.81, *p* = 0.058, $\eta_{\text{p}}^{2}$ = 0.087. Importantly, the Group × Condition interaction effect was significant, *F*_(1, 40)_ = 4.55, *p* = 0.039, $\eta_{\text{p}}^{2}$ = 0.102. Simple effect analysis revealed that children with ASD had lower *late eye-preference index* in the HAI condition than that in the LAI condition, *F*_(1, 40)_ = 8.35, *p* = 0.006, $\eta_{\text{p}}^{2}$ = 0.173, while TD children had a similar *late eye-preference index* in the HAI and LAI conditions, *F*_(1, 40)_ = 0.02, *p* = 0.898, $\eta_{\text{p}}^{2}$ < 0.001 (Figure 2D).

### Correlations Between Autism Symptoms and Eye Movement Indices

We correlated the ADOS total severity of children with ASD with the main eye-movement indices. No significant correlations were found, all *p*s > 0.05.

## Discussion

The primary aim of the present study was to test whether attention to eyes in children with ASD depended on the type of competing non-social stimuli (HAI vs. LAI objects) that were also present. Using the preferential looking paradigm, we revealed that attention to eyes was influenced by competing objects in both children with ASD and TD children, such that a decreased visual preference for the eyes when they were paired with HAI objects compared to LAI objects was observed. However, the modulation effects were different for the two groups in the early and late attention.

First, we found a reduced overall preference for the eyes when eyes were paired with HAI objects relative to LAI objects in both children with ASD and TD children. Second, with regard to the early and late attentional components, it was observed that all groups showed an initial higher probability of attending to the eyes when LAI objects were presented than High objects. After first initiating to the eyes, children with ASD showed higher sustained viewing preference to the eyes when LAI objects were presented, whereas this effect was absent in the TD group. After first looking at the objects, children with ASD were also more likely to show a higher probability of attending to the eyes when LAI objects were presented. In contrast, this trend was not significant for TD children.

These findings suggested that attention to eyes was somewhat contextually dependent in children with ASD, so that presence of HAI relative to LAI objects would reduce the relative salience of eyes to a greater degree. There are two types of salience that will attract social attention (37). One is social salience, which will drive the top-down volitional attention. The other one is physical salience (e.g., color, contrast, and motion), which will reflexively trigger the attention in a bottom-up way. In general, we may expect physical salience to primarily influence initial orienting to eyes, whereas top-down control will dominate the late attention phase [e.g., (38)]. In the current study, low-level stimulus properties (e.g., contrast, color, luminance, shape, and size) were not controlled across different objects (i.e., HAI and LAI objects), like many previous studies (15, 16, 22). Thus, it is difficult to conclude whether the object content alone or content and low-level physical properties best explains the modulation effect of CI on initial attention to eyes. However, late attention, especially the late eye-preference index, is more likely to reflect children's motivation or interest in viewing the eyes because the physical salience of objects may lose its control in guiding attention in the late phase after viewing the objects. Furthermore, if the physical salience of objects plays a role in biasing the attention in the late phase, we would expect the non-social objects to modulate the late eye-preference index in TD children like the results of the prioritization index. Together, our study implies that HAI objects can occupy visual attention in both early and late attention phases, therefore depriving visual experiences with eyes in children with ASD. Future studies with a sophisticated design could further test the contribution of bottom-up and top-down attention to eyes. Greater care should also be taken to control the low-level stimulus properties in the future.

Our data from the TD group indicated that the modulation effects of CI on attention to the eyes were not specific to children with ASD—TD children also showed some similar effects. Previous studies presenting a single face or a face embedded in a social context have found that children with ASD always showed less eye-looking time than TD children (39, 40). In contrast to these studies, we only included eye regions to control other facial features' impact on eye-preference. Besides, we only used one type of emotion (fear) in eyes, which removed social salience conveyed by human expression. These manipulations might significantly reduce the social significance of the eyes, resulting in a reduced motivation to process the eyes in TD children and thus the absence of group differences in the eye preference. Another possibility is that we only included one competing object to pair with the eyes, whereas in previous studies, multiple competitive objects [e.g., mouth and nose within a face, or toys and body within a social scene; (39, 40)] were present, which may exacerbate the eye avoidance in children with ASD. Finally, the chosen HAI objects (i.e., cars) might also be highly attractive to TD boys, which reduced the group difference. Future studies exploring how the attention to eyes in girls might be modulated by CI are recommended.

The modulation of HAI objects on gaze behavior is still informative for future clinical research and the development of interventions focusing on improving eye contact in ASDs. A number of interventions have tried to enhance the attention of individuals with ASD for socially relevant elements in a scene (41–43). Our study, from another perspective, suggests that in an intervention situation, making the non-social objects less salient could be used to increase social preference as a complementary approach. The presence of LAI objects could increase ASD children's propensity to look at the eyes first, heighten their interest in maintaining engagement with eyes after first looking at them, and improve their late looking preference to the eyes after first looking at the objects. However, it should be noted that we only used fearful eyes as stimuli, which was a limitation of our study. In reality, we usually look at a child in a non-fearful way. These settings could limit the generalizability of the current findigns. Thus, the implications for intervention should be taken with care, and future studies should test the generality of our findings to other expressions.

Another limitation in our study is that HAI objects we chose for children with ASD might also be interesting to TD children. Usually, HAI objects in ASD tend to be less attractive to TD children and are often idiosyncratic. Future studies using idiosyncratic HAI objects can replicate and extend our results.

In conclusion, we found that visual preference for eyes is influenced by competing objects in children with ASD—visual preference for eyes was reduced when HAI objects were present compared to when LAI objects were present. Our study not only helps us understand some factors that impact attention to eyes but also has implications for interventions aiming at improving eye contact in children with ASD.

## Data Availability Statement

The raw data supporting the conclusions of this article will be made available by the authors, without undue reservation.

## Ethics Statement

The studies involving human participants were reviewed and approved by The Association for Ethics and Human and Animal Protection in School of Psychological and Cognitive Sciences, Peking University. Written informed consent to participate in this study was provided by the participants' legal guardian/next of kin.

## Author Contributions

QW: conceptualization, methodology, software, writing- original draft preparation, formal analysis, and visualization. SPH: formal analysis, resources, investigation, and software. CS: investigation and writing–review and editing. TL: investigation. CML: investigation and conceptualization. YW: investigation and conceptualization. LY: conceptualization, writing–review and editing, supervision, and funding acquisition. All authors contributed to the article and approved the submitted version.

## Conflict of Interest

The authors declare that the research was conducted in the absence of any commercial or financial relationships that could be construed as a potential conflict of interest.

## Publisher's Note

All claims expressed in this article are solely those of the authors and do not necessarily represent those of their affiliated organizations, or those of the publisher, the editors and the reviewers. Any product that may be evaluated in this article, or claim that may be made by its manufacturer, is not guaranteed or endorsed by the publisher.

## References

[1] American Psychiatric Association. Diagnostic and Statistical Manual of Mental Disorders. 5th ed. Washington: American Psychiatric Press (2013). 10.1176/appi.books.9780890425596

[2] Mandy WP Skuse DH. Research review: what is the association between the social-communication element of autism and repetitive interests, behaviours and activities? J Child Psychol Psychiatry. (2010) 49:795–808. 10.1111/j.1469-7610.2008.01911.x 18564070

[3] Lam KSL Bodfish JW Piven J. Evidence for three subtypes of repetitive behavior in autism that differ in familiality and association with other symptoms: evidence for three subtypes of repetitive behavior in autism. J Child Psychol Psychiatry. (2008) 49:1193–200. 10.1111/j.1469-7610.2008.01944.x 19017031 PMC3709850

[4] Lee S Odom SL Loftin R. Social engagement with peers and stereotypic behavior of children with autism. J Posit Behav Interv. (2007) 9:67–79. 10.1177/1098300707009002040128218869

[5] Loftin RL Odom SL Lantz JF. Social interaction and repetitive motor behaviors. J Autism Dev Disord. (2008) 38:1124–35. 10.1007/s10803-007-0499-5 18064552

[6] Richler J Huerta M Bishop SL Lord C. Developmental trajectories of restricted and repetitive behaviors and interests in children with autism spectrum disorders. Dev Psychopathol. (2010) 22:55–69. 10.1017/S0954579409990265 20102647 PMC2893549

[7] Happé F Ronald A. The ‘fractionable autism triad': a review of evidence from behavioural, genetic, cognitive and neural research. Neuropsychol Rev. (2008) 18:287–304. 10.1007/s11065-008-9076-8 18956240

[8] Happé F Ronald A Plomin R. Time to give up on a single explanation for autism. Nat Neurosci. (2006) 9:1218–20. 10.1038/nn1770 17001340

[9] Lord C Jones RM. Annual Research Review: re-thinking the classification of autism spectrum disorders. J Child Psychol Psychiatry. (2012) 53:490–509. 10.1111/j.1469-7610.2012.02547.x 22486486 PMC3446247

[10] Leekam SR Prior MR Uljarevic M. Restricted and repetitive behaviors in autism spectrum disorders: a review of research in the last decade. Psychol Bull. (2011) 137:562–93. 10.1037/a0023341 21574682

[11] Black MH Chen N Iyer KK Lipp OV Bölte S Falkmer M. Mechanisms of facial emotion recognition in autism spectrum disorders: Insights from eye tracking and electroencephalography. Neurosci Biobehav Rev. (2017) 80:488–515. 10.1016/j.neubiorev.2017.06.016 28698082

[12] Speer LL Cook AE McMahon WM Clark E. Face processing in children with autism: effects of stimulus contents and type. Autism. (2007) 11:265–77. 10.1177/1362361307076925 17478579

[13] Tang J Falkmer M Horlin C Tan T Vaz S Falkmer T. Face recognition and visual search strategies in autism spectrum disorders: amending and extending a recent review by Weigelt et al. PLoS ONE. (2015) 10:e0134439. 10.1371/journal.pone.0134439 26252877 PMC4529109

[14] Frazier TW Strauss M Klingemier EW Zetzer EE Hardan AY Eng C. A meta-analysis of gaze differences to social and nonsocial information between individuals with and without autism. J Am Acad Child Adolesc Psychiatry. (2017) 56:546–55. 10.1016/j.jaac.2017.05.005 28647006 PMC5578719

[15] Sasson NJ Elison JT Turner-Brown LM Dichter GS Bodfish JW. Brief Report: Circumscribed attention in young children with autism. J Autism Dev Disord. (2011) 41:242–7. 10.1007/s10803-010-1038-3 20499147 PMC3709851

[16] Sasson NJ Touchstone EW. Visual attention to competing social and object images by preschool children with autism spectrum disorder. J Autism Dev Disord. (2014) 44:584–92. 10.1007/s10803-013-1910-z 23918441

[17] Wang Q Hu Y Shi D Zhang Y Zou X Li S. Children with autism spectrum disorder prefer looking at repetitive movements in a preferential looking paradigm. J Autism Dev Disord. (2018) 48:2821–31. 10.1007/s10803-018-3546-5 29589273

[18] Wang Q Lu L Zhang Q Fang F Zou X Yi L. Eye avoidance in young children with autism spectrum disorder is modulated by emotional facial expressions. J Abnorm Psychol. (2018) 127:722–32. 10.1037/abn0000372 30335441

[19] Dichter GS Richey JA Rittenberg AM Sabatino A Bodfish JW. Reward circuitry function in autism during face anticipation and outcomes. J Autism Dev Disord. (2012) 42:147–60. 10.1007/s10803-011-1221-1 22187105 PMC8624275

[20] Sasson NJ Dichter GS Bodfish JW. Affective responses by adults with autism are reduced to social images but elevated to images related to circumscribed interests. PLoS ONE. (2012) 7:e42457. 10.1371/journal.pone.0042457 22870328 PMC3411654

[21] Elison JT Sasson NJ Turner-Brown LM Dichter GS Bodfish JW. Age trends in visual exploration of social and nonsocial information in children with autism. Res Autism Spectr Disord. (2012) 6:842–51. 10.1016/j.rasd.2011.11.005 22639682 PMC3358817

[22] Harrop C Jones D Zheng S Nowell SW Boyd BA Sasson N. Sex differences in social attention in autism spectrum disorder. Autism Res. (2018) 11:1264–75. 10.1002/aur.1997 30403327 PMC7468514

[23] Faul F Erdfelder E Lang A-G Buchner A. G^*^ Power 3: a flexible statistical power analysis program for the social, behavioral, biomedical sciences. Behav Res Methods. (2007) 39:175–91. 10.3758/BF03193146 17695343

[24] Lord C Risi S Cook EH Leventhal BL DiLavore PC. The Autism Diagnostic Observation Schedule—Generic: a standard measure of social and communication deficits associated with the spectrum of autism. J Autism Dev Disord. (2000) 30:205–23. 10.1023/A:1005592401947 11055457

[25] Lord C Rutter M Couteur A. Autism Diagnostic Interview-Revised: a revised version of a diagnostic interview for caregivers of individuals with possible pervasive developmental disorders. J Autism Dev Disord. (1994) 24:659–85. 10.1007/BF02172145 7814313

[26] Wechsler D. Wechsler Intelligence Scale for Preschool and Primary Children–Fourth CN Edition, 4th Edn. King-May Company (2014)b.

[27] Wechsler D. Wechsler Intelligence Scale for Children–Fourth CN Edition, 4th Edn. King-May Company (2014)a.

[28] Gotham K Pickles A Lord C. Standardizing ADOS scores for a measure of severity in autism spectrum disorders. J Autism Dev Disord. (2009) 39:693–705. 10.1007/s10803-008-0674-3 19082876 PMC2922918

[29] Tottenham N Borscheid A Ellertsen K Marcus DJ Nelson CA. Categorization of facial expressions in children and adults: establishing a larger stimulus set. Poster Presented at the Annual Meeting of the Cognitive Neuroscience Society. San Francisco, CA (2002).

[30] Barnard-Brak L Abby L Richman DM Chesnut S. Facial emotion recognition among typically developing young children: a psychometric validation of a subset of NimStim stimuli. Psychiatry Res. (2017) 249:109–14. 10.1016/j.psychres.2016.12.049 28092789

[31] Houston JR Pollock JW Lien M-C Allen PA. Emotional arousal deficit or emotional regulation bias? An electrophysiological study of age-related differences in emotion perception. Exp Aging Res. (2018) 44:187–205. 10.1080/0361073X.2018.1449585 29578840

[32] Wang Q Hoi SP Wang Y Song C Li T Lam CM. Out of mind, out of sight? Investigating abnormal face scanning in autism spectrum disorder using gaze-contingent paradigm. Dev Sci. (2019) 23:e12856. 10.1111/desc.12856 31081980

[33] Kleberg JL Högström J Nord M Bölte S Serlachius E Falck-Ytter T. Autistic traits and symptoms of social anxiety are differentially related to attention to others' eyes in social anxiety disorder. J Autism Dev Disord. (2016) 47:3814–21. 10.1007/s10803-016-2978-z 28000078 PMC5676829

[34] Nyström P Bölte S Falck-Ytter T Team E. Responding to other people's direct gaze: alterations in gaze behavior in infants at risk for autism occur on very short timescales. J Autism Dev Disord. (2017) 47:3498–509. 10.1007/s10803-017-3253-7 28871495 PMC5633639

[35] Olsen A. The Tobii I-VT Fixation Filter. Algorithm Description. Stockholm: Tobii Technology (2012).

[36] Holmqvist K Nyström M Andersson R Dewhurst R Jarodzka H Van de Weijer J. Eye Tracking: A Comprehensive Guide to Methods and Measures. Oxford: OUP Oxford (2011).

[37] Laidlaw KE Risko EF Kingstone A. A new look at social attention: Orienting to the eyes is not (entirely) under volitional control. J Exp Psychol Hum Percept Perform. (2012) 38:1132–43. 10.1037/a0027075 22686696

[38] Langton SRH Law AS Burton AM Schweinberger SR. Attention capture by faces. Cognition. (2008) 107:330–42. 10.1016/j.cognition.2007.07.012 17767926

[39] Jones W Klin A. Attention to eyes is present but in decline in 2-6-month-old infants later diagnosed with autism. Nature. (2013) 504:427–31. 10.1038/nature12715 24196715 PMC4035120

[40] Yi L Fan Y Quinn PC Feng C Huang D Li J. Abnormality in face scanning by children with autism spectrum disorder is limited to the eye region: Evidence from multi-method analyses of eye tracking data. J Vis. (2013) 13:227–32. 10.1167/13.10.5 23929830 PMC3739407

[41] Alvares GA Chen NT Notebaert L Granich J Mitchell C Whitehouse AJ. Brief social attention bias modification for children with autism spectrum disorder. Autism Res. (2019) 12:527–35. 10.1002/aur.2067 30632321

[42] Amaral C Mouga S Simões M Pereira HC Bernardino I Quental H. A feasibility clinical trial to improve social attention in autistic spectrum disorder (ASD) using a brain computer interface. Front Neurosci. (2018) 12:e477. 10.3389/fnins.2018.00477 30061811 PMC6055058

[43] Turner-Brown LM Perry TD Dichter GS Bodfish JW Penn DL. Brief Report: feasibility of social cognition and interaction training for adults with high functioning autism. J Autism Dev Disord. (2008) 9:1777–84. 10.1007/s10803-008-0545-y 18246419 PMC2646378

